# Prevalence and longitudinal impact of kinesiophobia on outcomes following ACL reconstruction in adolescents and young adults

**DOI:** 10.1002/jeo2.70591

**Published:** 2025-12-10

**Authors:** Emma van der Schoor, Robin Voskuilen, Kim Derks, Martijn Dietvorst, Rob Janssen, Marieke van der Steen

**Affiliations:** ^1^ Department of Orthopaedic Surgery and Trauma Máxima Medical Centre Eindhoven the Netherlands; ^2^ Department of Health & Sports Fontys University of Applied Sciences Eindhoven the Netherlands; ^3^ Department of Biomedical Engineering, Eindhoven University of Technology Orthopaedic Biomechanics Eindhoven the Netherlands; ^4^ Department of Orthopaedic Surgery and Trauma Catharina Hospital Eindhoven the Netherlands

**Keywords:** adolescent, anterior cruciate ligament reconstruction, fear of movement, knee function, return to sports

## Abstract

**Purpose:**

The aim of this study was to longitudinally investigate the prevalence and impact of high‐level kinesiophobia in adolescents and young adults undergoing anterior cruciate ligament (ACL) reconstruction.

**Methods:**

In this retrospective cohort study conducted January 2018–January 2022, patients aged 16–21 undergoing ACL reconstruction were assessed before, and at 3 and 12 months postoperatively. Baseline characteristics were extracted from medical records. Kinesiophobia (Tampa Scale for Kinesiophobia, TSK‐17), subjective knee function (International Knee Documentation Committee, IKDC), readiness to return to sport (RTS) (ACL‐return to sport after injury, ACL‐RSI), and activity level (Tegner activity scale) were collected via an online questionnaire. Patients were categorized into high or low level kinesiophobia using a 37‐point cut‐off. Change in prevalence of kinesiophobia was analysed using the *χ* ^2^ test. Univariate logistic regression identified predictors of a high level of kinesiophobia at 12 months after ACL reconstruction. To explore the impact of kinesiophobia, between‐group differences in patient‐reported outcome measures over time were tested using repeated measures analysis of variance.

**Results:**

Data of 98 patients were available. Preoperatively, the prevalence of high kinesiophobia was 63.3%, decreasing to 37.8% at 3 months and to 26.7% at 12 months. Preoperative kinesiophobia was the only significant predictor of postoperative kinesiophobia (*p* = 0.002). The high kinesiophobia group scored significantly lower on IKDC (*p* < 0.001) and ACL‐RSI (*p* < 0.001). Both groups improved over time on Tegner (*p* < 0.001), IKDC (*p* < 0.001) and ACL‐RSI (*p* < 0.001).

**Conclusions:**

High kinesiophobia is common in adolescents and young adults undergoing ACL reconstruction. Kinesiophobia decreased the most in the first three months after reconstruction. In the group with high kinesiophobia, the knee function and readiness to RTS were significantly lower. This significant difference persists over time, despite a significant improvement in both groups. This study emphasizes the need to understand and address psychological characteristics in ACL rehabilitation.

**Level of Evidence:**

Level III.

**Abbreviations**
95% CI: 95% confidence interval
ACL: anterior cruciate ligament
ACL‐RSI: anterior cruciate ligament return to sport after injury scale
BMI: body mass index
BPTB: bone–patellar–tendon–bone
IKDC: International Knee Documentation Committee
IQR: interquartile range
LET: lateral extra‐articular tenodesis
PROMs: patient‐reported outcome measures
RTS: return to sport
TSK‐17: Tampa Scale for Kinesiophobia

## INTRODUCTION

Anterior cruciate ligament (ACL) rupture is a severe injury for young individuals. According to the World Health Organization (WHO), individuals between 16 and 21 years of age should be grouped as adolescents and young adults [43]. Over the last decade, incidence rates within this particular population have been rising [38, 48]. An ACL rupture has an impact on both social as well as physical functioning of adolescents, for example, they might lose the ability to participate in sports activities [29]. Current management consists of either conservative treatment or ACL reconstruction followed by physiotherapy [1]. Treatment aims to restore knee function, reduce further damage (e.g., re‐rupture/further meniscal damage), and prepare for return to sport (RTS) [1]. In recent years, a shift occurred from conservative treatment towards early operative management to prevent further damage, whilst keeping skeletal immaturity in mind [22]. A meta‐analysis and systematic review by Kay et al. reported a 90% RTS rate in adolescents after ACL reconstruction, yet return to a competitive level ranges widely from 41% to 81% [22, 40]. Moreover, re‐rupture rates in adolescents are high and range from 13% to 34% after ACL reconstruction, compared to 6% in adults [1, 22, 25, 47]. Thus, it is important to investigate factors that affect successful outcomes after ACL reconstruction in the adolescent population.

Several functional factors, such as injury to surgery time or the number of giving away episodes, affect the outcome after ACL reconstruction [3]. Furthermore, psychological aspects such as catastrophizing, depression, frustration, and fear of re‐injury are mentioned for their potential negative impact on the rate of recovery, RTS, and re‐injury rates after ACL reconstruction [39]. Fear of re‐injury is mentioned by 50% of the adolescents as the reason for not returning to sport [32]. Fear of re‐injury is part of a larger concept of kinesiophobia. Kinesiophobia is defined as ‘the fear of movement as a result of a feeling of susceptibility to pain or re‐injury’ [10]. Kinesiophobia is part of the ‘fear avoidance model’ composed of a complex vicious circle of catastrophizing, depression, and pain‐related disability leading to fear avoidance [24]. Kinesiophobia influences important outcomes such as gait, knee disability, and activity level in adults [3, 4, 27, 32, 37]. Additionally, kinesiophobia is proposed as a possible reason for the elevated risk of rupture in the contralateral leg [37]. In adults, the prevalence of high levels of kinesiophobia was 69.2% before surgery, which reduced to 43.1% after 3 months and to 30.8% after 12 months [45]. However, the prevalence and true impact of kinesiophobia in adolescents and young adults with an ACL rupture remain unknown. Paterno et al. suggested that kinesiophobia in younger individuals is associated with being less active, having less symmetrical strength, and being at higher risk for re‐rupture [40]. Considering the impact of kinesiophobia in adults, further assessment of the prevalence and impact of kinesiophobia in adolescents is necessary, as they may respond differently.

The aim of this study was to longitudinally investigate the prevalence of high‐level kinesiophobia. A secondary aim was to determine whether baseline kinesiophobia level (high vs. low) influenced patient‐reported outcomes following ACL reconstruction. This study hypothesizes that the prevalence of high levels of kinesiophobia will decrease over time following ACL reconstruction, and that patients with high baseline kinesiophobia will report worse outcomes on patient‐reported outcome measures (PROMs) at 3 and 12 months postoperatively compared to those with low baseline kinesiophobia.

## METHODS

### Study design

This retrospective analysis was part of a larger observational cohort study on outcomes of ACL reconstructions.

### Participants and sample size

All participants were recruited at Máxima Medical Center between January 2018 and January 2022. Patients were eligible if they were between 16 and 21 years of age at the time of reconstruction, had sufficient understanding of the Dutch language to complete the questionnaires, and were scheduled for primary ACL reconstruction [21]. The following exclusion criteria were applied: failure to complete the kinesiophobia questionnaire at baseline, prior ipsilateral ACL revision surgery, reconstruction with an allograft or objection to the use of their clinical data for research purposes.

### Data procedure

Data were collected as part of routine care at three time points: presurgery (T0), 3 months postoperatively (T3) and 12 months postoperatively (T12). Two experienced orthopaedic surgeons (R. J. and M. D.) who were blinded to the research question performed all ACL reconstructions. Baseline characteristics, including age, sex, body mass index (BMI), and injury side, were extracted from patients' medical records and entered into Research Manager (Research Manager, the Netherlands) by two independent researchers (E. S. and K. D.). PROMs were collected online via a digital data platform (Onlineproms; Interactive Studios). In order to enhance response rates, patients received a reminder to fill out the questionnaires via the digital platform and received the questionnaires on paper via mail in case of no response. Data analysis was performed by one researcher (E. S.).

### Prevalence of kinesiophobia

Kinesiophobia was assessed using the Tampa Scale for Kinesiophobia (TSK‐17), a 17‐item questionnaire, with a maximum of 68 points. The TSK is nowadays commonly used to assess kinesiophobia in ACL populations [6, 30]. The TSK‐17 has demonstrated good internal consistency (*α* = 0.79) and test–retest reliability (intraclass correlation coefficient [ICC] = 0.90, standard error of the mean [SEM] = 2.75) in patients with ACL ruptures [17]. A cut‐off score of 37 points has been established in a large cohort of patients with low back pain, with a score of ≤37 indicating low kinesiophobia and >37 indicating high kinesiophobia [46]. Although this cut‐off was established in low back pain patients, it has been widely applied across ACL research [7, 14, 30, 44]. Using this cut‐off maintains compatibility with the existing literature and facilitates potential clinical implications [20, 35]. The level of kinesiophobia measured at baseline (T0) was used to divide participants into those with high levels and those with low levels of kinesiophobia, prior to the ACL reconstruction. The same cut‐off was applied at 3 and 12 months to assess changes in kinesiophobia over time.

### Outcome of treatment

The outcome of treatment was primarily assessed by means of subjective knee function, while secondary outcomes included activity level and readiness to RTS at 3 and 12 months. Subjective knee function was measured with the Dutch adult version of the 2000 International Knee Documentation Committee (IKDC) subjective knee form [16]. The IKDC is a 10‐item questionnaire with a maximum score of 100 points, where higher scores indicate a higher level of activity with lower levels of symptoms. The IKDC demonstrated a good internal consistency (*α* = 0.92) and test–retest reliability (ICC = 0.95–0.96) [16, 18]. Furthermore, the questionnaire has been shown to be responsive in patients with ACL injuries and able to distinguish between patients who are able and unable to RTS [19, 28].

Activity level was measured with the Dutch–Tegner activity scale, which measures activity levels based on sport and work activities [12]. The Tegner activity scale ranges from 0 to 10 points, with higher scores representing a higher level of activity. It has shown good internal consistency (ICC = 0.97) in both adolescents and adults [12].

Readiness to RTS was measured with the Dutch version of the anterior cruciate ligament RTS after injury scale (ACL‐RSI) [44]. The ACL‐RSI consists of 12 items that evaluate emotion, confidence in performance, and risk appraisal. The maximum score is 100 points, with lower scores indicating a higher level of negative feelings towards RTS. The ACL‐RSI has good internal consistency (Cronbach's *α* = 0.94) and no floor or ceiling effects [44].

### Statistical analysis

The outcome variables of both groups were explored for normality and outliers using the Shapiro–Wilk test. Depending on the normality of the distribution, data were presented as means and their standard deviations or as medians and interquartile ranges (IQR). If more than 5% of data were missing for any outcome variable, the data were excluded pairwise, following verification that patients with and without missing data were comparable. All data were analysed using SPSS (version 26; IBM Corp.). For all analyses, the significance level was set at a *p* < 0.05.

For the primary aim of this study, patients were classified as high or low level kinesiophobia according to the cutoff point (37 points) of the TSK‐17 at baseline, 3 and 12 months postoperatively [7, 14, 44]. Changes in kinesiophobia distribution over time were analysed using the *χ* ^2^ test with Bonferroni correction. In addition, univariate logistic regression was performed to identify which variables predict the high level of kinesiophobia group at 12 months. Variables that were included in the univariate analyses were gender, days till surgery, BMI, age, kinesiophobia at baseline, Tegner activity scale at baseline, ACL‐RSI score at baseline and IKDC score at baseline [45]. Subsequently, a multivariate analysis was performed with the significant variables (*p* < 0.05) from the univariate analysis. The predictive capacity of the multivariate model was assessed with Nagelkerke *R* ^2^ [33, 41].

For the secondary aim, to test the differences in outcome on the PROMs (IKDC, Tegner and ACL‐RSI) at baseline, 3 months and 12 months between baseline high and low levels of kinesiophobia groups, a one‐way repeated measures analysis of variance (ANOVA) was used. Mauchly′s sphericity test was used to test equality of variances. If significant (*p* < 0.05), Greenhouse–Geisser was used. Furthermore, when a significant interaction between time and kinesiophobia group was observed, Bonferroni post hoc tests were conducted to determine which time points or group comparisons differed significantly.

## RESULTS

### Study population

**Figure 1:**
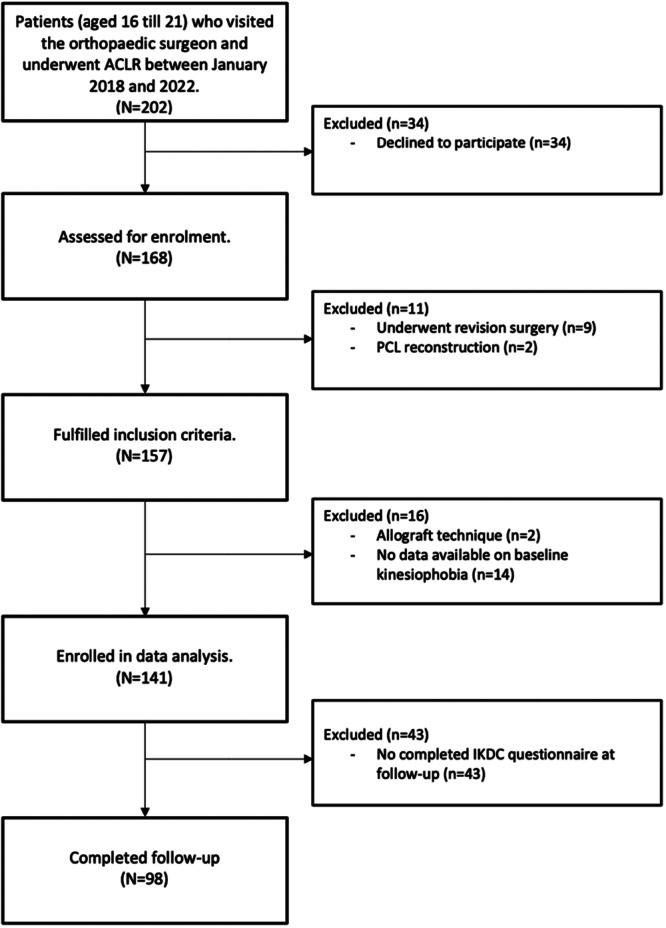
Flow diagram of participants in the study. ACLR, anterior cruciate ligament reconstruction; IKDC, International Knee Documentation Committee; PCL, posterior cruciate ligament.

In total, 202 adolescents and young adults were scheduled for an ACL reconstruction. Of these patients, 168 (83%) were willing to participate. Following the inclusion and exclusion criteria, 27 patients (16%) were excluded (see Figure 1). Twelve months following reconstruction, 30.5% (*n* = 43) did not complete the follow‐up questionnaires. These participants with missing data were equally distributed over those with high levels and low levels of kinesiophobia at baseline (*p* > 0.05). Participants with missing data were comparable to the potential study population on all baseline values (*p* > 0.05, see Supporting Information: File S1). As no differences were found in presurgery patient and injury characteristics, further analyses were performed on patients with complete data at 12 months following ACL reconstruction. Hence, a total of 98 patients were used as the study population. An overview of the baseline characteristics is shown in Table 1, both groups were similar presurgery (*p* > 0.05, see Table 1).

**Table 1:** Baseline patient characteristics.

Variables	Low‐level kinesiophobia (TSK ≤ 37)	High‐level kinesiophobia (TSK > 37)	*p*
	*N* = 36	*N* = 62	
Age (years)			
Median	19.0	18.5	ns^a^
IQR	[17.0–20.0]	[17.0–20.0]	
Sex (male)			
No. (percentage)	21 (58)	32 (52)	ns
BMI (kg/m^2^)			
Median	21.9	22.4	ns
IQR	[20.4–23.6]	[21.0–25.7]	
Injury side (left)			
No. (percentage)	21 (58)	34 (55)	ns
Noncontact trauma			
No. (percentage)	32 (89)	54 (87)	ns
Previous knee surgery			
No. (percentage)	6 (18)	9 (18)	ns
Time till surgery (days)			
Median	144	188	ns
IQR	[64–356]	[117–358]	
Additional knee damage^b^			
None	18 (50)	39 (63)	ns
Meniscal damage	12 (33)	27 (43)	ns
Collateral ligament damage	9 (25)	8 (13)	ns
Bone microfracture or avulsion	0 (0)	3 (5)	ns
Graft of reconstruction technique^c^			
Hamstring	31 (86)	51 (82)	ns
Hamstring + LET	5 (14)	7 (11)	ns
Quadriceps + LET	0 (0)	3 (5)	ns
BPTB + LET	0 (0)	1 (2)	ns

Abbreviations: BMI, body mass index; BPTB, bone–patellar–tendon–bone; IQR, interquartile range; LET, lateral extra‐articular tenodesis; TSK, Tampa Scale for Kinesiophobia.

^a^ Nonsignificant if *p* value > 0.05.

^b^ Presented as a number (percentage within the respective kinesiophobia group); multiple aspects could be possible within one patient.

^c^ Presented as a number (percentage within the respective kinesiophobia group).

### Prevalence of kinesiophobia

**Figure 2:**
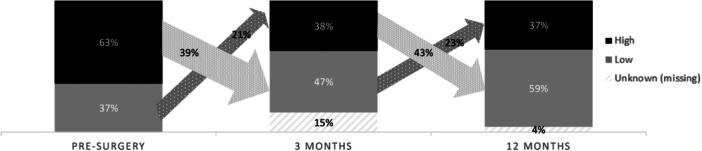
Prevalence of kinesiophobia in adolescents (16–21 years) preoperatively, 3 and 12 months following reconstruction (*n* = 98).

Presurgery, 63.3% (*n* = 62) of the study population displayed high levels of kinesiophobia (see Figure 2). After 3 and 12 months, prevalence of high‐level kinesiophobia decreased significantly to 37.8% (*n* = 37) and 36.7% (*n* = 36) (*p* = 0.002; *p* < 0.001). Importantly, Figure 2 illustrates that these changes were not uniform across patients; some transitioned from high to low kinesiophobia, whilst others switched from low to high levels. Specifically, 39% (*n* = 24/62) of patients with high‐preoperative kinesiophobia shifted to the low group at 3 months postsurgery, while 21% of those who initially reported low kinesiophobia switched into the high kinesiophobia group. A similar pattern was seen between 3 and 12 months; 43% (*n* = 19/37) of the patients with high kinesiophobia improved and transitioned to the low group, whereas 23% of those with low kinesiophobia developed high levels.

### Predictors of postoperative kinesiophobia

The univariate analysis identified two significant predictors of kinesiophobia group status at 12 months: preoperative kinesiophobia level (*p* < 0.001) and ACL‐RSI (*p* = 0.020). All other variables were not significant (see Table 2). In the subsequent multivariate analysis, which included the two significant predictors, only preoperative kinesiophobia remained a significant predictor (*p* = 0.002). The model predicted 23.0% of the variance in kinesiophobia group status at 12 months.

**Table 2:** Predictors of high‐level kinesiophobia 12 months following reconstruction.

	Univariate analysis	*p*	Multivariate analysis	*p*
	Odds ratio [95% CI]			
Age	0.823 [0.629;1.075]	ns*		
Gender	1.015 [0.441;2.339]	ns*		
BMI	0.076 [0.935;1.245]	ns*		
Days till surgery	0.000 [0.999;1.001]	ns*		
TSK‐17	1.139 [1.064;1.219]	<0.001	1.135 [1.049;1.227]	0.002
IKDC	0.996 [0.974;1.017]	ns*		
Tegner Activity Scale	1.200 [0.963;1.495]	ns*		
ACL‐RSI	0.976 [0.956;0.997]	0.020	0.998 [0.973;1.023]	ns*

Abbreviations: 95% CI = 95% confidence interval; ACL‐RSI, ACL‐readiness to return to sport after injury; BMI, body mass index; IKDC, International Knee Documentation Committee; TSK‐17, Tampa scale for kinesiophobia.

* Nonsignificant, if *p* value > 0.05.

### Secondary outcomes

**Figure 3:**
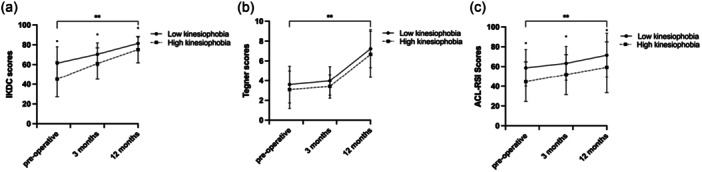
Differences in PROMs preoperatively, 3 months and 12 months following reconstruction. (a) IKDC, (b) Tegner and (c) ACL‐RSI. Data (*n* = 98) were expressed as mean values ± SD. ACL‐RSI, ACL‐readiness to return to sport after injury; IKDC, International Knee Documentation Committee; PROMs, patient‐reported outcome measures; SD, standard deviation. *Significantly different between low kinesiophobia compared to high kinesiophobia at individual timepoints (*p* < 0.05). **Significantly different over time, independent of group.

An overview of patient‐reported outcome variables, comparing groups with high and low preoperative levels of kinesiophobia at 12 months following ACL reconstruction, is shown in Figure 3.

At baseline, IKDC scores were significantly higher in the low kinesiophobia group compared to the high kinesiophobia group (difference = 16.4, *p* < 0.001) (Figure 3a). This difference persisted at both 3 months (difference = 9.3, *p* < 0.001) and 12 months following reconstruction (difference = 6.3, *p* < 0.001). Both groups showed an increase in IKDC scores over time (*p* < 0.001), significantly increasing across all time points (*p* < 0.001). However, no significant interaction effect (time × group) was found for IKDC scores (*p* = 0.072), indicating a similar improvement over time in both groups.

In terms of Tegner scores, there was no significant difference between the low and high kinesiophobia groups. Both groups, however, showed a similar increase over time (*p* < 0.001) (Figure 3b). Significant increases were observed from 3 to 12 months (*p* < 0.001) and from baseline to 12 months following reconstruction (*p* < 0.001)

Preoperatively, the high‐level kinesiophobia group scored significantly lower on ACL‐RSI scores compared to the low kinesiophobia group (difference = 14.0, *p* < 0.001) (Figure 3c). This difference remained consistent at 3 months (difference = 11.4, *p* < 0.001) and 12 months (difference=12.3, *p* < 0.001). While both groups showed an increase in ACL‐RSI scores over time (*p* < 0.001), significantly changing across all timepoints (*p* < 0.001). No significant interaction effect was found (time × group) (*p* = 0.826).

## DISCUSSION

The most important finding was that a high level of kinesiophobia was present in more than half of the young patients before surgery. The high level of kinesiophobia decreases over time but remains present in more than one‐third of young patients at 12 months following ACL reconstruction. Baseline kinesiophobia is an important predictor of a high level of kinesiophobia after ACL reconstruction. Furthermore, patients with high preoperative kinesiophobia levels were associated with poorer subjective knee function and readiness to RTS.

The current study showed that the prevalence of kinesiophobia was high and decreased over time, similar to findings in adults [6, 44]. In addition, 36.7% of the adolescents and young adults displayed high‐level kinesiophobia at 12 months, which is slightly higher than in adults at 30.8% [18]. It should be noted that these previous findings in adults were often not based on longitudinal follow‐up of patients undergoing ACL reconstruction but rather on combining assessments of kinesiophobia at different time points [5]. This is only the third study with multiple time follow‐up up to one year after ACL reconstruction [15, 28]. Combining longitudinal data on kinesiophobia and outcomes related to ACL reconstruction reduces bias and increases the clinical utility of the findings. In line with the adult literature, preoperative levels of kinesiophobia were an important predictor for kinesiophobia 12 months following reconstruction [45]. Considering the important role of preoperative kinesiophobia, it seems beneficial to screen patients for their preoperative kinesiophobia level [40]. Furthermore, the largest decrease in kinesiophobia occurred within the first 3 months after reconstruction, indicating a relevant timeframe to treat kinesiophobia.

As a secondary aim, this study investigated the impact of preoperative kinesiophobia level on rehabilitation after ACL reconstruction. The subjective knee function scores and readiness to RTS were significantly lower in the high‐level kinesiophobia group. Although both groups showed functional improvements over time, the persistent disparity suggests that psychological factors may hinder perceived functional recovery [41]. Results of the current study are in line with previous literature in adults, suggesting that kinesiophobia is associated with poorer subjective knee function [27, 32] and improvement over time [15, 42] following ACL reconstruction. One study even demonstrated that preoperative kinesiophobia affects postoperative gait biomechanics four months after ACL reconstruction, which may be one of the reasons why psychological factors play such a significant role in rehabilitation outcomes [44].

Interestingly, the activity levels were not significantly different between the high and low levels of kinesiophobia groups. Over time, Tegner scores of both groups increased significantly, but there was no significant difference at 12 months postoperatively between high and low kinesiophobia groups. This contrasts with previous research, showing that kinesiophobia is related to lower activity levels [4, 39]. This could be due to the used outcome parameter to assess the patients' activity level. The Tegner activity scale has demonstrated ceiling effects in active young adults, whereas RTS‐oriented outcomes, such as the Marx scale, could provide clearer differences in further research [47]. Next to that, the 12‐month follow‐up time may be too early as RTS is not always completed or actively delayed at this time. As could be indicated by the larger deviation at 12 months [23, 34].

Successful outcome of an ACL reconstruction—especially RTS to preinjury level of competitive sports—depends on many factors such as graft type, functional performance, patient characteristics, and psychological aspects [2, 11, 38]. These psychological aspects include readiness to RTS, kinesiophobia, pain catastrophizing and low self‐efficacy [39]. According to the fear avoidance model, kinesiophobia, pain catastrophizing and disuse may lead to a complex circle that negatively influences recovery [24]. Therefore, the psychological well‐being of the adolescents and young adults should be monitored preoperatively and intra‐recovery, and if necessary, additional treatment should be offered [39]. A recent study by Murray et al. reported that increasing positive psychological awareness within a supportive rehabilitation environment may improve rehabilitation experiences and RTS rates [47].

Considering the high prevalence and impact of high‐level kinesiophobia on the outcome of ACL reconstruction, it seems worthwhile to prevent or address high levels of kinesiophobia during medical management. Several treatments currently exist and seem to reduce kinesiophobia. These treatments included kinesiotaping, gradual exposure and multiple cognitive behavioural therapy techniques (such as controlled breathing, relaxation and goal setting) [8, 9, 13]. In addition, physiotherapists should be aware of kinesiophobia; by addressing physical and psychological issues, they can help overcome kinesiophobia and aid (p)rehabilitation [30]. However, further research is needed to better understand the direction of the relationship between factors influencing kinesiophobia and outcomes after an ACL injury. This will aid the development and evaluation of possible interventions for addressing kinesiophobia.

Certain limitations need to be considered when interpreting the findings of this study. Firstly, despite multiple invitations to fill out the questionnaires, loss to follow‐up was 30.4%. However, as the data was gathered during usual care, there were no incentives for the patients to participate. This likely contributed to the loss to follow‐up at 12 months. Missing data analysis showed no differences from the study cohort, with power remaining sufficient [26].

Secondly, when considering the course of kinesiophobia over time, it might be relevant to identify additional patient groups. There are patients who preoperatively have high levels of kinesiophobia but cross over to the low‐level group after ACL reconstruction. This study made groups solely based on the baseline level of kinesiophobia to assess the impact on clinical outcomes. This was chosen according to a validated cut‐off point and in consultation with clinicians, to identify possible treatment targets during the preoperative period and the first months after medical management. The finding that the preoperative level of kinesiophobia was a predictor of kinesiophobia 12 months after ACL reconstruction supports this reasoning.

## CONCLUSION

High levels of kinesiophobia are common in adolescents and young adults undergoing an ACL reconstruction. The prevalence decreased the most in the first three months after reconstruction. Patients with high kinesiophobia had worse subjective knee function and readiness to RTS than those with low kinesiophobia, and this difference remained even 12 months after surgery. This study emphasizes the need to further establish the role of psychological characteristics in ACL rehabilitation. Addressing psychological issues before reconstruction or in the first months of rehabilitation might improve clinical outcomes.

## AUTHOR CONTRIBUTIONS

**Emma van der Schoor**: Data acquisition; analyses and interpretation; drafting the manuscript. **Robin Voskuilen**: Interpretation of the data and drafting the manuscript. **Kim Derks**: Data acquisition and analysis; revision of the manuscript. **Rob Janssen**: Conception and design of the study; data interpretation and drafting the manuscript. **Martijn Dietvorst**: interpretation of data and revision of the manuscript. **Marieke van der Steen**: Conception and design of the study; data analysis and interpretation; drafting the manuscript. All authors read and approved the final manuscript.

## CONFLICT OF INTEREST STATEMENT

The authors declare no conflicts of interest.

## ETHICS STATEMENT

The local medical ethical committee declared that this study did not meet the criteria as stated by the Medical Research Involving Human Subjects Act (WMO) and the local committee approved the conduct of this study (METC 2018‐162; Máxima Medical Centre). Consent of patients or their legal guardians for the scientific use of data obtained in the process of usual care is obtained prior to treatment. Patients were given the opportunity to change their consent during their regular care visit or change it within the online hospital environment. Patients who made an objection against using their clinical data for research purposes were left out of the analyses.

## Supporting information

Supplementary Information

## Data Availability

The datasets used and/or analysed during the current study are available from the corresponding author on reasonable request.
