# Supplementary material for: Prevalence and longitudinal impact of kinesiophobia on outcomes following ACL reconstruction in adolescents and young adults
Source: J Exp Orthop. 2025 Dec 10;12(4):e70591. doi: 10.1002/jeo2.70591 (PMC12690398; doi:10.1002/jeo2.70591)
Supplement: Supplementary file 1 — Supplementary Information [file JEO2-12-e70591-s001.docx]

**Additional File 1: Incomplete data analysis**

*Table, differences in baseline values between incomplete patient data and study population at baseline*

| Variables | Total population (n=98) | Participants with missing IKDC at 12 months. | P |
| --- | --- | --- | --- |
|  | **N=98** | **N=43** |  |
| Group level at baseline |  |  |  |
| High level kinesiophobia | 62 | 25 | 0.564 |
| Low level kinesiophobia | 36 | 18 | 0.564 |
| Age |  |  |  |
| Median | 19.0 | 19.0 | 0.533 |
| IQR^a^ | [17.0-20.0] | [18.0-20.0] |  |
| Sex^b^ |  |  |  |
| No. (percentage) | 55 (56) | 26 (60) | 0.677 |
| BMI^c^ |  |  |  |
| Median | 22.2 | 23.3 | 0.365 |
| IQR | [20.7-24.30] | [20.6-24.9] |  |
| Injury side^d^ |  |  |  |
| No. (percentage) | 54 (55) | 17 (40) | 0.089 |
| Trauma mechanism^e^ |  |  |  |
| No. (percentage) | 85 (87) | 38 (88) | 0.789 |
| Previous knee surgery |  |  |  |
| No. (percentage) | 14 (14) | 10 (26) | 0.289 |
| Time till surgery |  |  |  |
| Median | 170 | 160 | 0.385 |
| IQR | [90-358] | [90-330] |  |
| Additional knee damage |  |  |  |
| None | 41 (42) | 19 (44) | 0.795 |
| Meniscal damage | 39 (40) | 21 (49) | 0.317 |
| Collateral ligament damage | 18 (18) | 7 (16) | 0.765 |
| Bone fracture or avulsion | 3 (3) | 2 (4) | 0.638 |
| IKDC |  |  |  |
| Median | 48.3 | 50.6 | 0.984 |
| IQR | [36.5-64.4] | [39.1-58.6] |  |
| Tegner Activity scale |  |  |  |
| Median | 3.0 | 3.0 | 0.823 |
| IQR | [2.0-4.0] | [2.0-4.0] |  |
| ACL-RSI |  |  |  |
| Median | 50.0 | 50.0 | 0.547 |
| IQR | [33.9-65.2] | [40.8-70.0] |  |
| TSK |  |  |  |
| Median | 41.0 | 40.0 | 0.461 |
| IQR | [36.0-46.0] | [34.0-46.0] |  |

*^a^IQR = interquartile range. ^b^Sex presented as number of males. ^c^BMI = Body Mass Index in kg/m2. ^d^injury side presented as number of left-sided injury. ^e^Trauma mechanism presented as number of non-contact trauma. ^f^Time to surgery in days.^g^Additional damage presented as number (percentage of total group).^h^Graft technique used as number (percentage). ^i^LET = Lateral extra-articulair tendonese. ^j^BPTB = bone-patellar-tendon-bone. ^k^IKDC = International Knee documentation Committee. ^l^ACL-RSI = ACL-Readiness to return to sport after injury. ^m^TSK-17 = Tampa scale for kinesiophobia *Non-significant if p-value >0.05*
